# Maternal and infant correlates of postpartum depression: a study from low socioeconomic communities in Karachi, Pakistan

**DOI:** 10.1186/s12884-026-08894-9

**Published:** 2026-03-04

**Authors:** Gul Afshan, Sadia Parkar, Nadia Mazhar, Samreen Memon, Sonila Ali, Zahra Hoodbhoy, Sidra Kaleem Jafri

**Affiliations:** Department of Paediatrics and Child Health, Aga Khan University, Karachi, Pakistan

**Keywords:** Postpartum depression, Maternal mental health, Low-income communities, Psychosocial stressors, Intimate partner violence

## Abstract

**Background:**

Postpartum depression (PPD) is a significant community issue impacting maternal health, child growth and development and family life. In Pakistan, its prevalence is notably high, particularly in socioeconomically disadvantaged settings, however, there is still limited community-based research done in examining its multiple contributing factors.

**Objective:**

To estimate the point prevalence of PPD among three groups of mothers at 1, 3, and 6 months postpartum and to identify maternal, socioeconomic, and psychosocial correlates in low-income peri-urban communities of Karachi, Pakistan.

**Methods:**

A cross-sectional analysis was conducted among 268 mothers of infants with ages of 1, 3, and 6 months, in the low socioeconomic districts of Karachi. Depressive symptoms were measured using the Edinburgh Postnatal Depression Scale (EPDS) with a country specific cut-off of 14 and above being the symptoms of clinically significant importance. Structured questionnaires were used to gather data on sociodemographic and obstetric and psychosocial characteristics. Data collected between July 2022 and December 2024. Poisson regression models were used to estimate crude prevalence ratios, adjusted prevalence ratios (aPR) with 95% confidence intervals (CIs).

**Results:**

The overall prevalence of possible PPD was 29.1% (95% CI: 23.7–34.5). Multivariable analysis identified significant associations of PPD with child’s male gender (aPR = 1.474, 95% CI:1.003–2.167), middle-born children (aPR = 2.104, 95% CI:1.119–3.956), intimate partner violence (aPR = 3.430, 95% CI:1.566–7.509), maternal employment (aPR = 2.706, 95% CI:1.468–4.987), household income (aPR = 0.259, 95% CI:0.088–0.761), and previously breastfeeding of two children (aPR = 0.413, 95% CI: (0.205–0.830) and three children (aPR = 0.181, 95% CI: (0.082–0.396). Additionally, significant intimate partner violence and household income interaction was observed (aPR = 7.049, 95% CI:1.714–28.982).

**Conclusion:**

This paper examined maternal and infant predictors of postpartum depression among low socioeconomic groups in Karachi, Pakistan. Our results highlight the necessity of context-based solutions to postpartum mental health in resource-constrained appropriations. Screening in communities, better houses, economical support and violence prevention helps are essential to decrease PPD load in resource constrained peri-urban environment.

## Introduction

Postpartum depression (PPD) is an important mental disorder marked by a complicated combination of physical, emotional, and behavioral alterations that happen in the initial year following birth. It is a significant health issue of the population because of its tremendous influence on the well-being of the maternal body, infant development, and family functioning [1, 2]. Globally, the prevalence of PPD ranges from 10 to 30% in high-income countries (HICs) [3, 4] and 18–31% in low- and middle-income countries (LMICs) [3, 5, 6]. For example, studies have reported postpartum depression prevalence of approximately 22% in India [7], 39.4% in Bangladesh [8], 32.8% in Saudi Arabia [9], and 34.8% in Iran [10]. These numbers underscore the uneven distribution of PPD in LMICs and emphasize the necessity of finding context-related risk factors, which make PPD develop and continue.

The Edinburgh Postnatal Depression Scale (EPDS) is the most widely used screening tool for assessing PPD in both high- and low-resource settings [11–13]. This is a self-report scale, a 10-item scale that is used to assess the severity of the depressive symptoms. The EPDS has demonstrated high sensitivity and specificity and has been culturally adapted for use in Pakistan, with typically employ cut-offs of ≥ 12–13 [14, 15]. It is easy to administer and is the best to use when conducting community-based research on low-literacy populations.

In Pakistan, prevalence estimates are consistently higher, ranging between 28% and 63%, with the burden unevenly distributed among the less socioeconomically advantaged groups [5, 16–18]. This large range in the estimates of prevalence is due to differences in measuring instruments, diagnostic cut-points and populations of study. These elevated rates are attributed to a complicated interplay of cultural, social, and structural factors such as the contradictory recommendations from extended family members, high strictness of cultural norms on gender role, low levels of literacy, poor health facilities, and widespread socioeconomic deprivation [17, 19].

Other correlations outlined by previous studies include low levels of maternal education, unplanned pregnancies, social lack of support, and exposure to intimate partner violence [18, 20]. Most of the current research in Pakistan has however been cross-sectional, hospital-based and only involved a single time point, giving an incomplete picture of how PPD changes in the early postpartum in vulnerability urban settings [16, 21]. Moreover, not much is available regarding the joint influence of socioeconomic, sociodemographic, and psychological issues in the formation of maternal mental health in low-income peri-urban settings, where structural and cultural issues increase risks. To fill these gaps, this study aims to estimate the prevalence of PPD at 1, 3, and 6 months postpartum and to identify its key correlates; including maternal characteristics, household socioeconomic status, and psychological stressors. By generating context-specific evidence, this research is aimed at providing context-specific evidence to inform interventions targeted to better maternal mental health and ultimately, to facilitate neurodevelopmental outcomes of resource-limited urban infants.

## Methodology

### Study design & participants

**Fig. 1:**
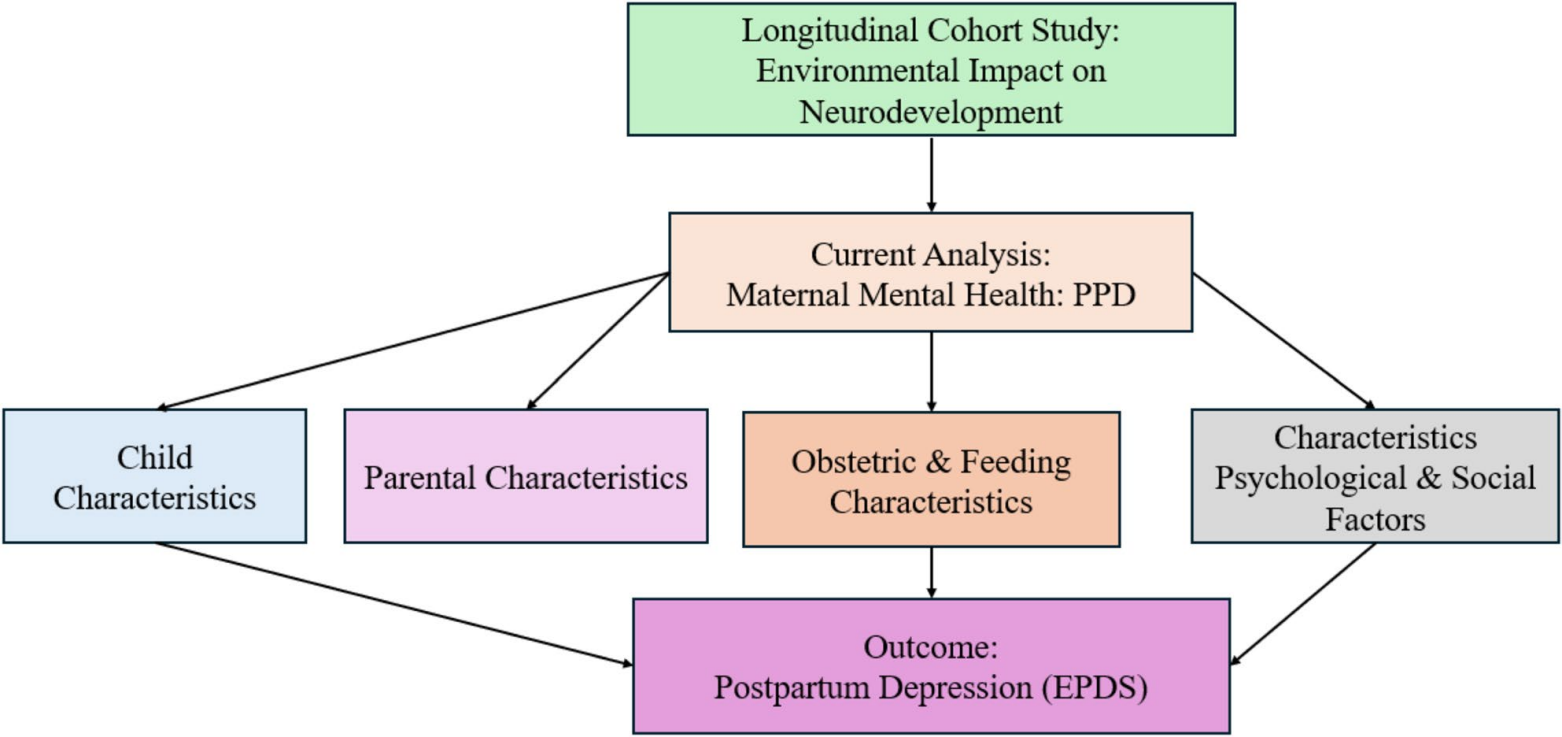
Conceptual framework showing the risk factors for postpartum depression (PPD)

This cross-sectional analysis is part of a larger longitudinal cohort study titled *“Integrating MRI Brain Imaging Studies to Learn the Neurodevelopmental Impact of Environment on the Early Life Years: A Longitudinal Cohort Study in a Low-Income Community”* [22]. The overarching study aims to examine the effects of early-life environmental exposures on neurodevelopment in children. The primary research problem is to longitudinally trace child growth and neurodevelopment paths during early infancy through 3.5 years of age using longitudinal MRI brain imaging and neurodevelopmental outcomes in Karachi. In this context, the present manuscript is based on the data of the above-mentioned ongoing research, which involves maternal mental health, with the consideration of the cross-sectional analysis of three independent groups of mothers having 1-month infants, 3 months of infants, and 6 months of infants. The enrollments were carried out in two peri-urban settlements of Karachi’s Rehri Goth and Ibrahim Hyderi, contiguous coastal villages on the outskirts of Karachi. Eligible participants, mothers of healthy infants (mother-infant’s dyads), were recruited from surveillance study “The Pregnancy Risk, Infants Surveillance, and Measurement Alliance” (PRISMA) [22]. These communities have minimal access to healthcare facilities and are attended to by primary healthcare facilities under Aga Khan University that offer routine maternal and child health care. A flow chart illustrating the study design and factors affecting maternal PPD is presented in Fig. 1.

### Eligibility criteria

The eligibility criteria for the main study and the current analysis include mothers of infants aged 1, 3 and 6 months who were permanent residents of either Rehri Goth or Ibrahim Hyderi and had delivered a live-born infant with no significant neonatal and maternal complications. Mothers of infants with any congenital anomalies, history of hypoxic-ischaemic insult, delayed cry, requiring oxygen, preterm or hospitalization were excluded. Mothers with conditions like pre-eclampsia, high blood pressure, or diabetes mellitus in pregnancy or ultrasound abnormalities (e.g., ventriculomegaly, renal agenesis, gastrointestinal structural anomalies) were excluded. Others that were also excluded were mothers who were on antidepressants or anti-epileptic drugs during pregnancy and fetal exposure to smoking (cigarettes).

### Sample size

**Fig. 2:**
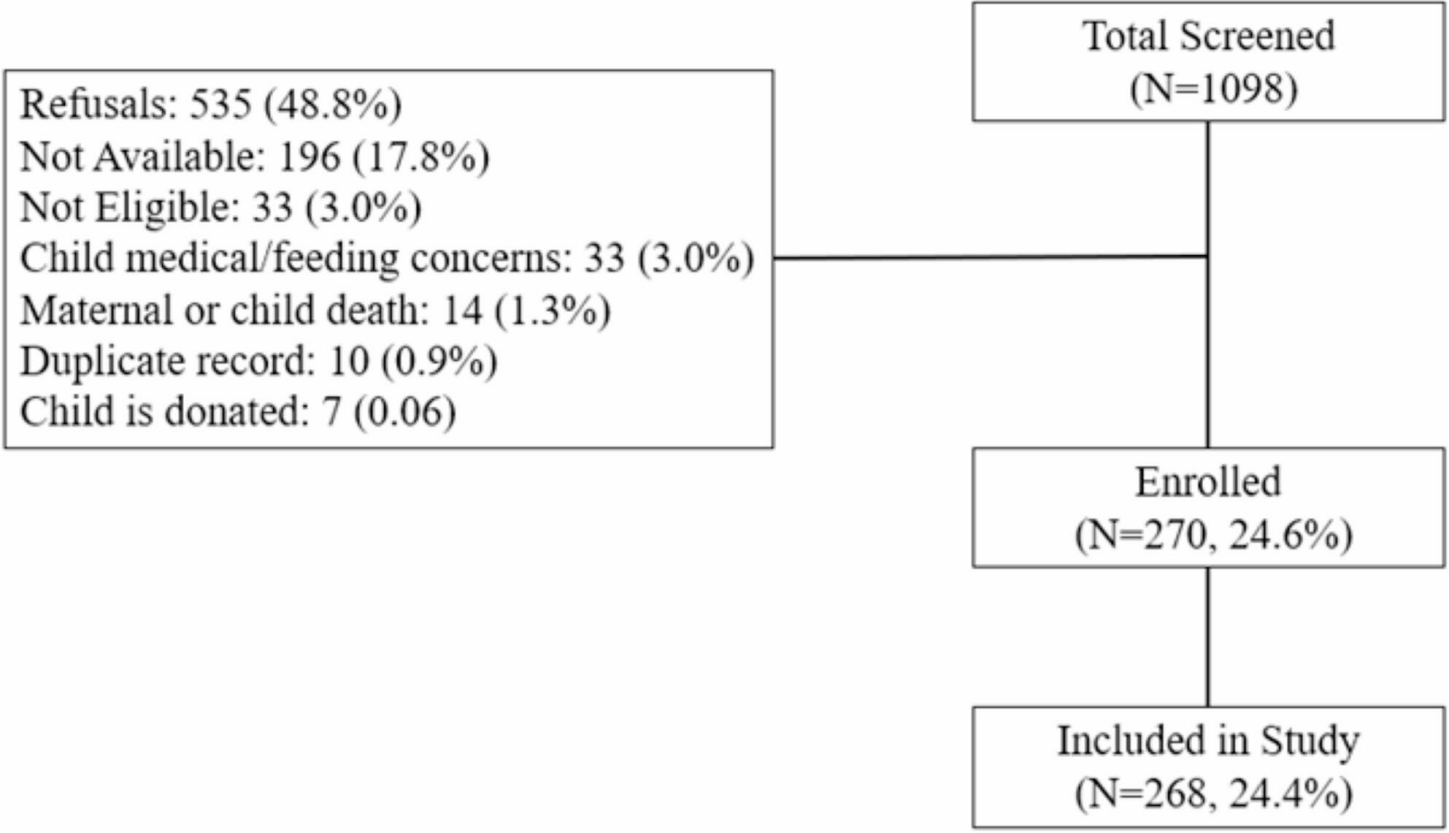
Flow diagram of participant recruitment and inclusion

The sample size in the original cohort was calculated on the basis of feasibility, informed by the capability to recruit and past infant neuroimaging studies and the estimated attrition of 40% in the study during longitudinal follow-up. Since the current analysis relies on existing baseline data of this current cohort, a formal priori calculation of sample size on postpartum depression was not done. Mothers who met the predefined eligibility criteria were enrolled consecutively from the parent study. Of 1,098 mothers screened, 828 were excluded. The most common reasons were refusal to participate (*n* = 535, 48.8%), unavailability after screening (*n* = 196, 17.8%), and failure to meet inclusion criteria (*n* = 33, 3.0%). A detailed flow diagram is provided in Fig. 2. Overall, at baseline, data were collected from 270 mother-infant dyads, however, PPD data were available for 268 mothers. Therefore, the final sample for the present study comprised 268 mother-infant dyads.

### Data collection methods

The enrolment-phase data was collected between July 2022 and December 2024. The main outcome variable, postpartum depression, was assessed using EPDS, which is a screening instrument that had been culturally adapted and validated in the Pakistani setting [14]. The EPDS was administered among three independent groups of mothers at 1, 3, and 6 months postpartum. The EPDS was administered by trained female research assistant in the local language during face-to-face interviews. The severity of depressive symptoms was categorized in four categories based on EPDS score: none or minimal depression (0–6), mild depression (7-13), moderate depression (14-19), and severe depression (20-28) [23] . For overall statistical analysis, possible depression (EPDS score ≥ 14) was considered [24, 25]. This cut-off is based on study showing optimal diagnostic performance against the Clinical Interview Schedule-Revised (CIS-R) with improved specificity and positive predictive value [24, 25]. EPDS was administered by trained female senior research assistants at study field site. In addition, a comprehensive case report form (CRF) was completed to collect detailed sociodemographic and socioeconomic information pertaining to both the infants and parents (both mother and father). Details of the covariables include:

### Covariables

The covariates analyzed in this study were grouped into four main categories.

#### Child demographics

This category included factors related to the child, such as gender, age (1 month, 3 months, or 6 months), birth order (firstborn, middle child (2–3)) or later born (≥ 4).

#### Parental demographics

Maternal age (≤ 18, 19–25, 26–30 and > 30 yrs) and paternal age (18–29, 30–39 and 40–60 yrs) were taken into consideration in the analysis. Education levels of both parents were categorized as no education/0 year, 1–8 years, and 9 + years. Employment status of mothers and fathers was also assessed, classified as employed or unemployed for father while employed and stayed-at-home for mothers. The BMI was classified underweight (> 18.5), Normal (18.5–25.0), overweight (25-29.9) and obese (> 30).

#### Obstetric and breastfeeding characteristics

This category included maternal obstetric and breastfeeding factors. Obstetric characteristics comprised maternal pregnancies during last 5 years categorized 1 pregnancy, 2 Pregnancies, 3–5 Pregnancies, sleep duration categorized as < 6 h, 6–8 h, and > 8 h per night. Maternal prior maternal breastfeeding experience were assessed by the number of children that had been breastfed before the index child with none, first, second, third, and more than three being the categories.

#### Psychological and social characteristics

The Hurt, Insult, Threaten, Scream (HITS) tool was used to measure psychological factors, which identified intimate partner violence. HITS was categorized as negative: <10, and positive: ≥ 10. Social characteristics included household-related variables such as house ownership (rented or owned by either parents), number of rooms in the household (single room, 2–3 rooms, or 4–7 rooms), monthly household income (0–177 USD and > 177 USD), and total household size (no of family member: 3–6, 7–10, 11–15, > 15).

### Ethical consideration

Ethics approval of the study was obtained from the Ethics Review Committee of AKU (ERC Number: 2022-7181-21831).

### Data Analysis

Maternal characteristics were presented in frequencies and percentages of categorical variables and means and Standard Deviation (SD) of continuous variables. Point Prevalence of the PPD was represented in percentage with 95% CI. Considering the high prevalence of the PPD, it was feasible to use the robust variance Poisson regression model for estimating the prevalence ratios [26]. In the bivariate model, each variable was used to test the association with PPD to calculate crude prevalence ratios (cPRs). Data completeness was assessed prior to analysis. There were no missing values for the primary outcome (EPDS score), and missingness across covariates was negligible (< 1%). The adjusted effect of the factors on PPD was assessed using Multivariable Poisson regression model modelling. Bivariate analysis variables with p-values were below 0.20 were included in the multivariate model. Moreover, a priori variables that were theoretically considered relevant according to existing literature were included in the adjusted model irrespective of statistical significance to reduce residual confounding. The multicollinearity of the variables was assessed using the generalized variance inflation factor (GVIF) adjusted for degrees of freedom, ${GVIF}^{\frac{1}{2.df}}$. Following prior literature, a VIF ≥ 2.5 is considered indicative of potential multicollinearity concerns; therefore, a corresponding threshold of ${GVIF}^{\frac{1}{2.df}}$≥ 1.58 (i.e., √2.5 ≈ 1.58) was used as the exclusion criterion [27]. All statistical analyses were conducted using RStudio (2025.05.0 + 496) with R (version 4.3.1).

## Results

### Study participants

A total of 268 mother-infant dyads were enrolled, with 39.9% (*n* = 107) infants of 1-month age, 39.2% (*n* = 105) of 3-months age group, and 20.9% (*n* = 56) at 6-months age. Gender distribution of infants of was almost the same, with 51.9% (*n* = 139) males and 48.1% (*n* = 129) females. The majority of mothers’ infants were middle-born infants (*N* = 119, 44.4%) (Table 2).

Most mothers were aged between 19 and 25 years (*n* = 148, 55.2%) while fathers were predominantly aged between 30 and 39 years (*n* = 121, 45.2%). Literacy rate was low, with nearly half of mothers (*n* = 125, 46.6%) and over two-fifths of fathers (*n* = 110, 41.0%) having no formal education. Mostly fathers were employed (*n* = 252, 94.0%) while mothers were stay-at-home (*n* = 247, 92.2%). Just over half of the mothers had a normal BMI (*n* = 139, 53.9%) (Table 3).

Regarding household and social characteristics, 72.4% (*n* = 194) mothers reported house ownership, and slightly more than half of the mothers lived in house sizes ranging between 2 and 3 rooms (*n* = 138, 51.5%). For most mothers, monthly household income was less than 177 USD (*n* = 238, 88.8%) (Table 5).

### Prevalence of PPD

Overall, 9.7% (*n* = 26, 95% CI: 6.6–13.2) mothers reported severe depression, while 19.4% (*n* = 52, 95% CI:14.7–24.1) were moderate, 39.9% mild (*n* = 107, 95% CI: 34.1–45.8) and 31.0% (*n* = 83, 95% CI: 25.4–36.5) reported minimal depression. The overall prevalence of possible postpartum depression (EPDS score ≥ 14) was approximately 29.1% (*n* = 78, 95% CI: 23.7–34.5). Prevalence rate was approximately same across 1-month (*n* = 29, 27.6%, 95% CI: 19.1–36.2), 3-months (*n* = 32, 29.9%, 95% CI: 21.2–38.6) and 6-months (*n* = 17, 30.4%, 95% CI: 18.3–42.4) post-partum period (Table 1).

**Table 1:** Prevalence of PPD at three-month period (*N* = 268)

Depression Severity	1-Month (*n* = 105)	3-Months (*n* = 107)	6-Months (*n* = 56)	Overall (*n* = 268)
None or minimal depression (0–6)	34 (32.4%) [23.4–41.3]	32 (29.9%) [21.2–38.6]	17 (30.4%) [18.3–42.4]	83 (31.0%) [25.4–36.5]
Mild depression (7–13)	42 (40.0%) [30.6–49.9]	43 (40.2%) [30.9–49.5]	22 (39.3%) [26.5–52.1]	107 (39.9%) [34.1–45.8]
Moderate depression (14–19)	18 (17.1%) [9.90–24.4]	22 (20.6%) [12.9–28.2]	12 (21.4%) [10.7–32.2]	52 (19.4%) [14.7–24.1]
Severe depression (20–30).	11 (10.5%) [4.6–16.3]	10 (9.3%) [3.8–14.9]	5 (8.9%) [1.5–16.4]	26 (9.7%) [6.6–13.2]
Possible Depression (≥ 14)	29 (27.6%) [19.1–36.2]	32 (29.9%) [21.2–38.6]	17 (30.6% [18.3–42.4]	78 (29.1%) [23.7–34.5]

### Factors associated with PPD

Mothers of male infants had a slightly higher PPD prevalence (*n* = 47, 33.8%) compared to mothers of female (*n* = 31, 24.0%), although this association was not statistically significant at the conventional 0.05 level (*p* = 0.079). Also, PPD prevalence was highest among later-born (birth order > 4) infants (*n* = 27, 30.3%), and middle-born (Birth order 2–3) (*n* = 36, 30.3%) as compare to mothers of firstborn (*n* = 15, 25.0%), while prevalence ratio was not significant (*p* > 0.05). Similarly, no significant association was reported PPD for infants age group (Table 2).

**Table 2:** Prevalence of PPD across child characteristics and its association using crude Prevalence Ratio (PR) (*N* = 268)

Variables	Overall	PPD prevalence		Crude PPD prevalence ratio	
	*N* (%)	*N* (%)	95% CI	PR (95% CI)	*p*-value
Child’s Gender					
Female	129 (48.1%)	31 (24.0%)	16.7%–31.4%	Reference	
Male	139 (51.9%)	47 (33.8%)	25.9%–41.7%	1.615 (0.949–2.777)	0.079
Birth Order					
Firstborn (1)	60 (22.3%)	15 (25.0%)	14.0%–36.0%	0.765 (0.36–1.588)	0.478
Middle Child (2–3)	119 (44.4%)	36 (30.3%)	22.0%–38.5%	0.996 (0.549–1.82)	0.989
Later born (4–13)	89 (33.2%)	27 (30.3%)	20.8%–39.9%	Reference	
Child’s Age					
1-month	105 (39.2%)	29 (27.6%)	19.1%–36.2%	Reference	
3-months	107 (39.9%)	32 (29.9%)	21.2%–38.6%	1.118 (0.616–2.034)	0.713
6-months	56 (20.9%)	17 (30.4%)	18.3%–42.4%	1.142 (0.554–2.316)	0.714

Table 3 presents the outcome of the crude PPD prevalence ratio for parental characteristics. PPD prevalence was high among mothers aged older than 30 years (*n* = 13, 31.0%) and mothers age between 19 and 25 years (*n* = 45, 30.1%). Meanwhile, 34.4% (*n* = 11) of mothers with PPD had partners aged 40–60 years, though association was not significant. Mothers with obesity (*n* = 10, 37.0%) had a higher prevalence ratio as compared to mothers with normal BMI (*n* = 36, 25.9%). PPD was highly common among mothers with no education (*n* = 39, 31.2%) and mothers who were employed (*n* = 10, 47.6%). A borderline significant association was observed between employed mothers and PPD (PR = 2.393, 95% CI: (0.956–5.929), *p* = 0.058). Also, 32.2% (*n* = 29) of mothers with PPD had partners with 1–8 years of education. PPD was higher among mothers with unemployed partners (*n* = 7, 43.8%). Nonetheless, analysis showed no significant associations between these parental characteristics and maternal PPD.

**Table 3:** Prevalence of PPD across parental characteristics and its association using crude Prevalence Ratio (PR) (*N* = 268)

Variables	Overall	PPD prevalence		Crude PPD prevalence ratio	
	*N* (%)	*N* (%)	95% CI	PR (95% CI)	*p*-value
Mother’s Age					
≤ 18	15 (5.6%)	4 (26.7%)	4.3%–49.0%	0.811 (0.196–2.894)	0.756
19–25	148 (55.2%)	45 (30.1%)	23.0%–37.8%	0.975 (0.471–2.098)	0.946
26–30	63 (23.5%)	16 (25.4%)	14.6%–36.1%	0.759 (0.319–1.823)	0.533
> 30	42 (15.7%)	13 (31.0%)	17.0%–44.9%	Reference	
Father’s Age					
18–29 yrs	115 (42.9%)	29 (25.2%)	17.3%–33.2%	0.644 (0.28–1.531)	
30–39 yrs	121 (45.2%)	38 (31.4%)	23.1%–39.7%	0.874 (0.389–2.047)	0.310
40-60Yrs	32 (11.9%)	11 (34.4%)	17.9%–50.8%	Reference	
Mother’s BMI					
Underweight	42 (15.7%)	14 (33.3%)	19.1%–47.6%	1.431 (0.666–2.985)	0.346
Normal	139 (53.9%)	36 (25.90%)	18.6%–33.2%	Reference	
Overweight	60 (22.4%)	18 (30.0%)	18.4%–41.6%	1.226 (0.619–2.379)	0.551
Obese	27 (10.1%)	10 (37.0%)	18.8%–55.3%	1.683 (0.687–3.964)	0.240
Mother’s Education Level					
No Education	125 (46.6%)	39 (31.2%)	23.1%–39.3%	Reference	
1–8 yrs	98 (36.6%)	27 (27.6%)	18.7%–36.4%	0.839 (0.465–1.497)	0.554
≥ 9	45 (16.8%)	12 (26.7%)	13.7%–39.6%	0.802 (0.364–1.685)	0.570
Father’s Education Level father					
No Education	110 (41.0%)	32 (29.1%)	20.6%–37.6%	Reference	
1–8 yrs	90 (33.6%)	29 (32.2%)	22.6%–41.9%	1.159 (0.632–2.122)	0.632
≥ 9	68 (25.4%)	17 (25.0%)	14.7%–35.3%	0.813 (0.403–1.599)	0.553
Mother’s Occupation					
Stayed-at-home	247 (92.2%)	68 (27.5%)	22.0%–33.1%	Reference	
Employed	21 (7.8%)	10 (47.6%)	26.3%–69.0%	2.393 (0.956–5.929)	0.058
Father’s Occupation					
Unemployed	16 (6.0%)	7 (43.8%)	19.4%–68.1%	Reference	
Employed	252 (94.0%)	71 (28.2%)	22.6%–33.7%	0.504 (0.181–1.46)	0.191

Mothers with only one pregnancy (*n* = 29, 30.8%) during the last 5 years had higher PPD prevalences as compared to mother with 2 or 3–5 pregnancies, though association was not significant. Mothers who breastfeed 3 children had less PPD (*n* = 29, 34.5%) compared to mothers who had breastfeed more than 3 children (PR = 0.306, 95% CI: (0.096–0.813), *p* = 0.026). Compared with mothers who slept more than 8 h (*n* = 20,62.0%), mothers sleeping less than 6 h per day had higher PPD prevalence (*n* = 24, 34.3%). However, no statistically significant association was found (*p* > 0.05) (Table 4).

**Table 4:** Prevalence of PPD across obstetric and feeding characteristics and its association using crude Prevalence Ratio (PR) (*N* = 268)

Variables	Overall	PPD prevalence		Crude PPD prevalence ratio	
	*N* (%)	*N* (%)	95% CI	PR (95% CI)	*p*-value
Total Pregnancies (5yrs)					
1 Preg.	94 (35.1%)	29 (30.8%)	21.5%–40.2%	1.338 (0.631–2.944)	0.455
2 Preg.	122 (45.5%)	36 (29.5%)	21.4%–37.6%	1.256 (0.61–2.697)	0.545
3–5 Preg.	52 (19.4%)	52 (25.0%)	13.2%–36.8%	Reference	
Previously breastfed					
None	47 (17.5%)	13 (27.7%)	14.9%–40.4%	0.725 (0.325–1.564)	0.420
One child	53 (19.8%)	17 (32.1%)	19.5%–44.6%	0.896 (0.426–1.851)	0.768
Two children	48 (17.9%)	14 (29.2%)	16.3%–42.0%	0.781 (0.356–1.666)	0.528
Three children	36 (13.4%)	5 (13.9%)	2.6%–25.2%	0.306 (0.096–0.813)	0.026*
More than 3	84 (31.3%)	29 (34.5%)	24.4%–44.7%	Reference	
Breastfeeding Frequency					
2 h per day	216 (80.6%)	59 (27.3%)	21.4%–33.3%	Reference	
4 h per day	18 (6.7%)	6 (33.3%)	11.6%–55.1%	0.752 (0.278–2.242)	0.585
Sleep Duration					
Less than 6 h	70 (26.1%)	24 (34.3%)	23.2%–45.4%	1.487 (0.733–3.045)	0.273
6 to 8 h	121 (45.2%)	34 (28.1%)	20.1%–36.1%	1.114 (0.588–2.148)	0.743
More than 8 h	77 (28.7%)	20 (25.9%)	16.2%–35.8%	Reference	

* *p*_value < 0.05

Mothers who screened positively for intimate partner violence had a higher prevalence of PPD (*n* = 34, 53.1%) with significant prevalence (PR = 4.121, 95% CI: (2.284–7.511), *p* < 0.001). Mothers living in a household with 2–3 rooms (PR = 0.487, 95% CI: (0.267–0.885), *p* = 0.018) had significantly low PPD as compared to mothers living in a single room house. Mothers with monthly household incomes above 177 USD had a lower prevalence of PPD (*n* = 5, 16.7%) compared to those earning ≤ 177 USD (*n* = 73, 30.7%), though impact was not significant (Table 5).

**Table 5:** Prevalence of PPD across Psychological and Social Factors and its association using crude Prevalence Ratio (PR) (*N* = 268)

Variables		PPD prevalence		Crude PPD prevalence ratio	
		*N* (%)	95% CI	PR (95% CI)	*p*-value
Intimate Partner Violence					
Negative	202 (75.4%)	44 (21.6%)	16.1%–27.5%	Reference	
Positive	66 (24.6%)	34 (53.1%)	39.5%–63.6%	4.121 (2.284–7.511)	< 0.001**
House Ownership					
Own	194 (72.4%)	57 (29.4%)	23.0%–35.8%	1.028 (0.573–1.890)	0.927
Rent	74 (27.6%)	21 (28.4%)	18.1%–38.7%	Reference	
Number of Room					
Single (1) Room	81 (30.2%)	31 (38.3%)	27.7%–48.9%	Reference	
2–3 room	138 (51.5%)	32 (23.2%)	16.1%–30.2%	0.487 (0.267–0.885)	0.018*
4–7 room	49 (18.3%)	15 (30.6%)	17.7%–43.5%	0.712 (0.329-1.5)	0.377
Monthly Household Income					
0-177 USD	238 (88.8%)	73 (30.7%)	24.8%–36.5%	Reference	
> 177 USD	30 (11.2%)	5 (16.7%)	3.3%–30.0%	0.452 (0.148–1.137)	0.119
Family size					
03–06	117 (43.7%)	38 (32.5%)	24.0%–41.0%	Reference	
07–10	92 (34.3%)	24 (26.1%)	17.1%–35.1%	0.734 (0.397–1.338)	0.316
11–15	44 (16.4%)	11 (25.0%)	12.2%–37.8%	0.693 (0.306–1.486)	0.359
> 15	15 (5.6%)	5 (33.3%)	9.5%–57.2%	1.039 (0.306–3.144)	0.947

* *p*_value < 0.05, ** *p*_value < 0.001

Mothers of the female children were associated with higher risk of PPD as compared to mothers of male children (aPR = 1.474, 95% CI: (1.003–2.167), *p* = 0.048). Similarly, childbirth order (2–3) was associated with higher odds of PPD (aPR = 2.104, 95% CI: (1.119–3.956), *p* = 0.021). Employed mothers showed higher likelihood of PPD prevalence as compared to stay-at-home (aPR = 2.706, 95% CI: (1.468–4.987), *p* = 0.001), indicating association between PPD and employed mothers. Intimate partner violence was also associated with higher prevalence of PPD (aPR = 3.430, 95% CI: (1.566–7.509), *p* = 0.002) when adjusted by other socio-demographic variables. Furthermore, a significant interaction between household income and IPV was observed (aPR = 7.049, 95% CI: (1.714–28.982), *p* = 0.007), indicating the combined effect of IPV and income. Higher household income (> 177 USD) was associated with lower odds of PPD compared to income level below 177 USD (aPR = 0.259, 95% CI: (0.088–0.761), *p* = 0.014). Mothers who previously breastfed two children (aPR = 0.413, 95% CI: (0.205–0.830), *p* = 0.013) and three children (aPR = 0.181, 95% CI: (0.082–0.396), *p* = 0.001) were associated with lower likelihood of PPD compare with mothers who had breastfeed more than 3 children (Table 6).

**Table 6:** Multivariate Model showing associated factors of PPD using adjusted prevalence ratios (aPR)

Variable	aPR (95% CI)	*p*-value
Gender		
Female	Reference	
Male	1.474 (1.003, 2.167)	0.048*
Birth Order		
Firstborn (1)	1.293 (0.419, 3.992)	0.655
Middle Child (2–3)	2.104 (1.119, 3.956)	0.021*
Later born (4–13)	Reference	
Father’s Age		
18–29 yrs	0.628 (0.347, 1.137)	0.124
30–39 yrs	0.832 (0.478, 1.448)	0.516
40-60Yrs	Reference	
Mother’s BMI		
Normal	Reference	
Obese	1.219 (0.673, 2.207)	0.514
Overweight	1.039 (0.675, 1.600)	0.861
Underweight	1.055 (0.588, 1.892)	0.859
Mother’s Employment		
Stayed-at-home		
Employed	2.706 (1.468, 4.987)	0.001**
Intimate Partner Violence		
Negative	Reference	
Positive	3.430 (1.566, 7.509)	0.002**
Intimate Partner Violence (Positive) × Mother Employment (Employed)	0.435 (0.14, 1.358)	0.152
Intimate Partner Violence (Positive) × Income (> 177 USD)	7.049 (1.714, 28.982)	0.007**
Intimate Partner Violence (Positive) × Sleep (less than 6 h)	0.486 (0.174, 1.352)	0.167
Intimate Partner Violence (Positive) × Sleep (6–8 h)	0.673 (0.261, 1.735)	0.412
Number of Room		
1 Room	Reference	
2–3 room	0.805 (0.500, 1.295)	0.370
4–7 room	1.099 (0.644, 1.876)	0.728
Monthly Household Income		
0-177 USD	Reference	
> 177 USD	0.259 (0.088, 0.761)	0.014*
Family size		
03–06	Reference	
07–10	0.695 (0.442, 1.092)	0.114
11–15	0.869 (0.458, 1.648)	0.667
> 15	1.235 (0.573, 2.662)	0.590
Total Pregnancies (5yrs)		
1 Preg.	Reference	
2 Preg.	1.009 (0.596, 1.707)	0.973
3–5 Preg.	0.681 (0.349, 1.327)	0.259
Number of children breastfed before this infant		
None	0.846 (0.276, 2.589)	0.769
One child	0.705 (0.355, 1.399)	0.317
Two children	0.413 (0.205, 0.830)	0.013*
Three children	0.181 (0.082, 0.396)	0.001**
More than 3	Reference	
Sleep Duration		
Less than 6 h	1.532 (0.792, 2.963)	0.205
6 to 8 h	0.944 (0.528, 1.690)	0.847
More than 8 h	Reference	

* *p*_value < 0.05, ** *p*_value < 0.001

## Discussion

The present study found that nearly 30% of mothers from low socioeconomic communities in Karachi experienced possible postpartum depression. The prevalence of depressive symptoms remained relatively stable across one, three, and six months group, with only modest variation. The key psychosocial and socioeconomic correlates of PPD include adjusted effect of child’s gender, birth order, household income, intimate partner violence, maternal employment, breastfeeding experience while unadjusted effect of number of rooms in household. Although not statistically significant, other potential risk factors that emerged from this study include maternal insufficient sleep, maternal obesity, and maternal education.

The prevalence rate reported in this study is consistent with previous research conducted in Pakistan, where prevalence rates typically range from 28% to 63% [5, 16–18] However, this prevalence is also similar to 18–31% prevalence in LMICs and 10–30% prevalence in HICs [3, 4]. A longitudinal study from a rural sub-district of Pakistan reported a marked decline in postpartum depression from 94% at three months to 76% at 6-months and 62% at 12-months postpartum [20], whereas another longitudinal study observed much lower but fluctuating proportions of postpartum depressive symptoms, ranging from approximately 3% to 9% across multiple postpartum time points up to 24 months after childbirth [28]. These variations in studies can be attributed to the study design, assessment tool for screening PPD, different cut-off and sociocultural differences [29].

Study analysis reported higher likelihood of PPD among mothers of male infants (aPR = 0.474). These outcomes are consistent with study in the context of UK, showing approximate 79% PPD prevalence among mothers of male infants [30]. However, study from India [31] reported nearly 4 times higher odds of PPD for mothers with female children (OR = 3.630). One possible explanation for these findings is fluctuations in hormone levels, such as higher levels of angiogenic factors and proinflammatory cytokines for male fetal, along with sociocultural concerns about raising boys, particularly in low-income communities [32]. Similarly, study showed a higher prevalence of PPD among mothers of children with lower birth order, specifically among mothers of middle child (birth order = 2–3). This association may be explained by the increased caregiving burden experienced by first-time mothers facing adjustment challenges and other maternal experience [25].

Our study identified a significant relationship between intimate partner violence and PPD. These findings align with study outcome by Necho et al. who reported significant impact of intimate partner violence on PPD (aPR OR = 6.500, 95% CI:1.980–15.850,) [33]. Consistent with existing evidence showing that intimate partner violence triggers psychological distress and neurological tension, while at the same time, it hinders coping mechanisms and emotional support, thus increasing susceptibility to depressive symptoms during the postpartum period [34].

This study also identified a significant interaction between household income and intimate partner violence, demonstrating that the combined effect of economic disadvantage and IPV was strongly associated with maternal depression. Though an increase in household income is broadly viewed as protective against maternal depression, the current study observed that the positive relation of intimate partner violence to PPD was stronger among higher income women. Likewise, this highlights the strong link between lower Socioeconomic Status (SES) and PPD by identifying socio-demographic factors such as a higher household income (aPR = 0.259) and number of rooms in the household as an associated factor of PPD (PR = 0.487). These findings are highly consistent with previous studies showing financial challenges as significant predictors of psychological vulnerability [35–37], such as Hairol et al. reported that mothers in Kuala Lumpur, Malaysia with low household income were twice likely to have PPD symptoms than those with higher incomes [35]. Similarly, according to study conducted by Gebregziabher et al. in Eritrea, East Africa, mothers who perceived their socioeconomic status (SES) as low were 13 times more likely to develop PPD as compared to the mothers who had good SES [38]. In our study, maternal employment was associated with higher likelihood of PPD. Likewise, Gebregziabher et al. showed that stay-at-home were 0.240 times less likely to develop PPD as compared to the employed mothers [39]. In low-income setting, maternal employment can indicate a socio-economic need, where women are involved in low paid employment besides doing the household and caring duties which may add up to psychological stress [40]. Moreover, inadequate social support, absence of maternity benefits and minimal work protections might increase the emotional burden [41]. Therefore, employment in such contexts represent socio-economic strain rather than psychosocial benefit.

Our study also reported that mothers’ higher breastfeeding experience showed a higher likelihood of postpartum depression. The relationship between breastfeeding and PPD is often considered complex, as women experiencing depression may find difficulty in breastfeeding, while women having trouble in breastfeeding may develop depression [42]. Though association was insignificant, this study also highlighted that reduced maternal sleep hours were associated with a higher prevalence of PPD. One of the studies conducted by McEvoy et al. also revealed that low sleep quality in the early postpartum was a predictor of depressive symptoms later in high-risk women [43]. Similarly, Zhang et al. reported 61.32% PPD among mothers with poor sleep [44]. Likewise, maternal obesity showed insignificant association with PPD. However previous studies showed that mothers with higher BMI are more vulnerable to psychological distress, such as Alzarooni et al. reported 36.3% women with PPD had obesity with 22.7% were overweight [45]. The relationship between obesity and depression can be seen as bidirectional where both conditions reinforcing each other through biological mechanisms such as neuroinflammation, dysfunction of the gut-brain axis, dysregulation of the hypothalamic-pituitary-adrenal axis and behavioral factors [46].

### Strengths and limitations

The key strength of the study is its community-based approach and inclusion of multidisciplinary factors to provide a comprehensive understanding of the possible associated risk factors of PPD.

The study also has limitations. The sample size of the study was limited to have adequate statistical power to reveal strong relations among various risk factors since the study was simply intended to examine the neurodevelopment of infants as opposed to maternal mental health outcomes. Overall, with 29.1% prevalence of PPD, the study may have been underpowered to detect associations, especially those based on less frequent exposures or interaction effects. Given the cross-sectional design, the outcome of the study limits the causal association between exposures and postpartum depression and offers only one time-point measure. Also, the present study was carried out in two coastal villages around Karachi and hence the results cannot be completely generalized to other areas of Pakistan or to the population with other socio-demographic, or cultural or health-care characteristics.

In addition, mothers who had obstetric complications were excluded to concentrate on comparatively healthy mothers in the community. As the established risk factors of postpartum depression include obstetric complications like pre-eclampsia, gestational hypertension, and diabetes mellitus, their omission can have resulted in an understatement of the actual prevalence of PPD. Thus, this exclusion criterion may affect the generalizability of findings for higher risk obstetric populations and tertiary care settings.

## Conclusion

This study examined maternal and infant correlates of postpartum depression among women living in low socioeconomic communities in Karachi, Pakistan. The findings highlight the importance of considering both maternal and infant-related factors when assessing postpartum mental health in resource-limited settings. Integrating brief postpartum depression screening into routine postnatal care delivered by community health workers may facilitate early identification and referral of at-risk mothers in similar communities. In resource-constrained peri-urban areas, improved housing situation, financial support, and violence prevention interventions play a crucial role in decreasing the burden of PPD. Strategic and effective intervention could be made more efficient by targeting those who are more likely to develop depression.

## Data Availability

The datasets used or/and analyzed during the current study are available from the corresponding author on reasonable request.

## Abbreviations

MRI: Magnetic Resonance Imaging
HICs: High-income countries
LMICs: Low- and middle-income countries
MINE: Maternal and environmental Impact assessment on Neurodevelopment in Early childhood years
PRISMA: Pregnancy Risk, Infants Surveillance, and Measurement Alliance
ERC: Ethics Review Committee of Aga Khan University
CRF: Comprehensive Case Report Form
BMI: Body Mass Index
HITS: Hurt, Insult, Threaten, Scream
USD: United States Dollar
SD: Standard Deviation
cPRs: Crude Prevalence Ratios
GVIF: Generalized Variance Inflation Factor
aPR: Adjusted Prevalence ratios
SES: Socioeconomic Status
